# How Dietary Diversity Enhances Hedonic and Eudaimonic Well-Being in Grazing Ruminants

**DOI:** 10.3389/fvets.2020.00191

**Published:** 2020-04-15

**Authors:** Matthew R. Beck, Pablo Gregorini

**Affiliations:** Faculty of Agriculture and Life Sciences, Lincoln University, Lincoln, New Zealand

**Keywords:** grazing, ruminants, animal welfare, taxonomical diversity, biochemical diversity

## Abstract

Ruminants evolved in diverse landscapes of which they utilized, by choice, a diverse arrangement of plants (grasses, forbs, and trees) for food. These plants provide them with both primary (carbohydrates, protein, etc.) and secondary (phenolics, terpenes, etc.) compounds (PPC and PSC, respectively). As no one plant could possibly constitute a “balanced-diet,” ruminants mix diets so that they can exploit arrangements of PPC to meet their individual requirements. Diet mixing also allows for ruminants to ingest PSC at levels, acquiring their benefits such as antioxidants and reduced gastrointestinal parasites, without overstepping thresholds of toxicity. Meeting dietary requirements is assumed to provide satisfaction through achieving positive internal status and comfort, thereby a sense of hedonic (happiness through pleasure) well-being. Furthermore, choice including dietary choice is a factor influencing well-being of ruminants in a manner akin to that in humans. Choice may facilitate eudaimonic (happiness through pursuit of purpose) well-being in livestock. Nutritional status plays an integral role in oxidative stress, which is linked with illness. Several diseases in livestock have been directly linked to oxidative stress. Mastitis, metritis, hypocalcaemia, and retained placenta occur in animals transitioning from dry to lactating and have been linked to oxidative stress and such a stress has likewise been linked to diseases that occur in growing livestock as well, such as bovine respiratory disease. The link between physiological stress and oxidative stress is not well-defined in livestock but is evident in humans. As dietary diversity allows animals to select more adequately balanced diets (improved nutrition), take advantage of PSC (natural antioxidants), and allows for choice (improved animal well-being) there is a strong possibility for ruminants to improve their oxidative status and thus health, well-being, and therefor production. The purposes of this review are to first, provide an introduction to oxidative and physiological stress, and nutritional status as effected by dietary diversity, with special attention to providing support and on answering the “how.” Second, to provide evidence of how these stresses are connected and influence each other, and finally discuss how dietary diversity provides a beneficial link to all three and enhances both eudaimonic and hedonic well-being.

## Introduction

Dietary diversity in ruminants has recently received considerable attention in the literature (1–8). Much of this work has focused on how dietary diversity can improve animal production by providing animals with the opportunity to choose and mix their diets. By doing so, the animals are better able to meet their individual requirements and self-medicate, acquiring nutraceuticals, pharmaceuticals, and prophylactic benefits associated with the ingestion of specific secondary compounds (PSC) at self-regulated safe levels of intake (9). Here we use the term well-being when discussing the subjective mental state of the animal and welfare as the animal state including well-being, health and the animals experience with their environment. In a comprehensive review article on dietary diversity and welfare, Manteca et al. (4) concluded that the improved nutritional status given by appropriate supply of plant primary compounds (PPC) and the improved health benefits by the PSC are indicative of the intimate relationship between dietary diversity and animal welfare. However, the benefits of dietary diversity on animal welfare have been discussed only as they relate to hedonic well-being. The word hedonic stems from the Greek word *hēdone*, meaning pleasure, and thus hedonic well-being is the balance between positive and negative emotions (10). Emotions are clusters of experiences related to health, fear, nutritional comfort, nutrient supply, and familiarity, as a few examples. Animals integrate those experiences, at different time scales forming either positive or negative emotions (11). Another concept of well-being, commonly applied to humans, is eudaimonia, which was first proposed by Aristotle. Eudaimonia stems from the Greek words *Eu*, for good, and *daimon*, for guardian. There are several definitions proposed for eudaimonia, but the one which we propose can best be applied to ruminants is one of function. We propose that eudaimonic well-being is achieved in livestock and other animals when they are able to pursue their potential (10). To that end, eudaimonic well-being is achieved when a subject achieves its *telos*, which is defined as a given purpose (12). Eudaimonic well-being has rarely been applied to livestock welfare but (12) proposed that an animal's *telos* is enshrined in the species' uniqueness which is genetically coded [see also (13)]. We propose that *telos* may also be considered as an individual trait and this is supported by individual animal personalities, by genetically related grazing personalities in ruminants (8, 14), and by the reduction of stress when choice is allowed (4, 15). Improved well-being by offering choice to animals both facilitates and provides evidence in support for eudaimonia and *telos* in livestock, as it has been suggested that without choice one cannot pursue their *telos* and thus achieve eudaimonic well-being (12). Even if the available options (e.g., dietary options) provided will only allow the animals to choose the least-worse option available for their individual needs, we argue eudaimonic well-being will be improved.

We hypothesize that merely providing choice would improve eudaimonic well-being in livestock; however, for dietary diversity to improve hedonic well-being there must first be some subsequent actions to increase pleasure or reduce negative experiences and thereby emotions. Such actions constitute responses to environmental stimuli that provoke oxidative stress, physiological stress, or reduced nutritional state of the animal. These three features of animal state are of interest with regard to welfare and hence production. Oxidative stress influences the pathophysiology of diseases, and its management has received much attention (16–19). Physiological stress including cortisol release is often used as an index of welfare (20), which in turn is linked to production and economic return (21). Appropriate nutrition for each respective class of livestock is obviously a major feature of every livestock production system.

**Figure 1:**
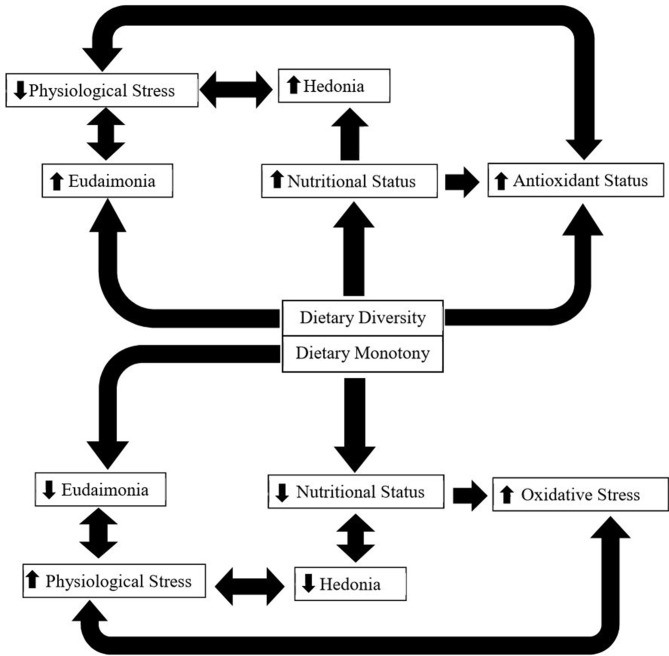
This figure shows a conceptual model depicting how dietary diversity vs. monotony may affect ruminants. Dietary diversity improves nutritional status, through ingesting complimentary plant compounds (primary and secondary); improves hedonic and eudaimonic well-being; decreases physiological stress; and improves antioxidant status, through the ingestion of plant secondary compounds that exhibit antioxidant effects. It is important to note that both nutritional status and physiological stress can also impact antioxidant status directly. On the other hand, dietary monotony may reduce nutritional status (by not allowing animals to mix a diet containing a balance of plant primary compounds) and hedonic (reduced nutritional status) and eudaimonic (loss of choice) well-being.

In this review we describe and explain how the influence of oxidative stress, physiological stress, and nutritional state influence well-being of grazing livestock as a response to taxonomic and biochemical diversity of the diet. We present a conceptual model (Figure 1) describing the interactive links between dietary diversity and animal state, resulting in positive effects on animal health and well-being (both hedonic and eudaimonic).

## Oxidative Stress

Oxidative stress is a state of imbalance between oxidants (e.g., reactive oxygen metabolites) and antioxidants [both enzymatic [e.g., superoxide dismutase] and non-enzymatic [e.g., vitamin E and glutathione]; (22, 23)]. The circulating level of oxidants is subject to homeostatic regulation but situations may occur in which the animal is exposed to stressors, such as high metabolic demand, gastrointestinal parasites, heat stress, and diseases (19), which cause the rate of production of oxidants to exceed the capacity of the homeostatic regulatory system. The remaining oxidants damage important biological molecules [including lipids, proteins, DNA, and RNA; (19)], which then lead to metabolic and pathological disorder (24).

An example of this is isoprostane production, which has similar actions as prostaglandins (e.g., prostaglandin F-2α). Prostaglandins are involved in the regulation of many physiological functions (e.g., pregnancy maintenance) and also in inflammation and immune responses (25). The key enzymes involved in the conversion of arachidonic acid to eicosanoids (e.g., prostaglandins) is cyclooxygenases (25). In cattle, prostaglandin F_2α_ are an important part of the estrus cycle as they cause luteolysis [degradation of the corpus luteum; (26)]. Prostaglandins are also important in the pathological manifestation of chemical or physical injury, in fact nonsteroidal anti-inflammatory drugs function by inhibiting prostaglandin synthesis, by blocking the cyclooxygenases (27). Similar compounds to prostaglandins, the isoprostanes, are generated independently of cyclooxygenase enzymes through the peroxidation of arachidonic acid by oxidants (28). Isoprostanes have been identified as a promising *in vivo* marker for oxidative stress and they have been found to have negative biological effects as they can bind to many of the same receptors as cyclooxygenase derived prostaglandins (28). These effects include vasoconstriction and airway constriction, and therefore may be pathophysiological mediators of oxidative damage (28). Thus, isoprostanes are formed through oxidants oxidizing a biological molecule (arachidonic acid) and subsequently inducing inflammatory responses to oxidative damage. We postulate that the damage to biological compounds leading to metabolic or pathological disorders and inflammation, such as arachidonic acid, would result in discomfort and subsequently reduce well-being. This is supported by some works who found a positive correlation between blood cortisol and isoprostane concentrations (29, 30). The integration of uncomfortable experiences leads to negative emotions, thus reducing hedonic well-being. The link between oxidative and physiological stress is discussed further below.

### Physiological Causes of Oxidative Stress

The following paragraphs provides a summary of the biochemical sources of oxidants, which is the “how” behind oxidative stress. This detail is important because it provides background information for understanding how biochemical (PSC) diverse diets reduce oxidative stress in grazing ruminants. We later describe how the improved antioxidant status of the animals would lead to enhanced hedonic well-being.

Oxidants are important in several physiological and biochemical reactions; consequently, they are well managed by the body. For example, Superoxide (${\text{O}}_{2}^{•-}$) is an oxidant produced in the mitochondria of mammalian cells, which is subsequently converted to H_2_O_2_ by mitochondrial superoxide dismutase (31, 32). This ${\text{O}}_{2}^{•-}$ is generated in the electron transport chain, with the majority being produced by complex I, and a negligible amount by complex III (32). These ${\text{O}}_{2}^{•-}$ are converted to H_2_O_2_, which exits the mitochondria to act as a redox signal to the cellular cytosol and the nucleus. Hydrogen peroxide, while still an oxidant, has a lower second order rate constant for reactions with biomolecules than other oxidants (e.g., hydroxyl radical or ${\text{O}}_{2}^{•-}$) and is therefore appropriate for redox signaling (33). These redox reactions are important in regulation of enzymes and transcription factors (33, 34). This elicits various cellular responses such as enzyme activity, substrate supply, and mitochondrial biogenesis (32, 35). Redox signaling shows how oxidant production, when under normal physiological functions, is necessary rather than negative.

Another physiological source of oxidants come from phagocytic cells removing foreign organisms. Reactive oxygen species are toxic to many microorganisms. When phagocytic cells (e.g., neutrophils) engulf bacteria there is an initial oxygen consumption (called oxidative burst) where an NADPH oxidase complex transfers electrons from NADPH to oxygen in order to generate superoxide. As some works have reported that superoxide does not kill bacteria, it is believed that additional secondary oxidants are generated and responsible for bacterial death (36). One important example of phagocytic cells to ruminants is neutrophils involvement in removing pathogens related to pneumonia such as *Mannheimia haemolytica*. Removal of these pathogens is reliant on active immunity and the innate immune function, such as neutrophils (37). Therefore, oxidant creation in this context is required by the body to remove foreign organisms and to maintain health and comfort through the relief from pain, and thus hedonic well-being.

The final source of oxidants to be discussed in this review occurs in the gastrointestinal tract. Halliwell et al. (38) proposed several sources of gastrointestinal derived oxidants. Firstly, foods will generally contain iron, often in the insoluble Fe^3+^ salt form. Gastric acid can solubilize ferric and metallic iron. The Fe^3+^ is then reduced to Fe^2+^, which is easier to absorb with stimulation by ascorbate. The oxidant, hydroxyl radical can be produced when ascorbate and Fe^2+^ are mixed, without H_2_O_2_ (Fenton Chemistry). Similar reactions can occur from Cu^2+^ and ascorbate (38). Other sources include: haem (from haem proteins), dietary lipids that undergo peroxidation, foods containing isoprostanes, oxidized cholesterol, nitrites, the gastrointestinal immune system, and oxidized phenolic compounds such as hydroxyhydroquinone [from coffee; (38)]. This may indicate that oxidized phenolic compounds from forage plants may act as oxidants. While information from the human literature is abundant, to our knowledge, few works have reported how important ruminant gastrointestinal derived oxidants are. One experiment measured antioxidant activity of rumen fluid and plasma from faunated or defaunated rumens [with or without protozoa; (39)]. It was found that faunated rumens had greater antioxidant activity than defaunated rumens in both ruminal fluid and in the plasma (39). Increased antioxidant capacity in the rumen leading to increased antioxidant capacity in the plasma would indicate that rumen fluid is important to the whole animal's antioxidant status. However, we are unaware of any research showing how this would translate to the lower gastrointestinal tract, but due to research on humans (see (38) for a review), we speculate that this is a significant source of oxidant production which requires further investigation.

### Defense Mechanisms Against Oxidative Stress

#### Enzymatic Defense Against Oxidative Stress

Antioxidant enzymes are a major intrinsic, or endogenous, oxidant defense. Superoxide dismutase (SOD) is found in the cytoplasm and the mitochondria of cells in the Cu-SOD and Mn-SOD forms, respectively (40). This enzyme converts the superoxide anion (which is highly radical) to hydrogen peroxide (H_2_O_2_; which is less radical). Glutathione peroxidase (GPx) is then responsible for converting H_2_O_2_ to water and oxidized glutathione. Catalase is another important antioxidant enzyme which converts H_2_O_2_ to water and O_2_. Glutathione reductase then “recycles” the oxidized glutathione by reducing it to its active form, reduced glutathione. This reaction occurs by oxidation of NADPH to NADP^+^ by GR (41).

As these enzymes are important in maintaining homeostasis of ruminants, their quantification in biological samples have been identified as a marker of oxidative stress (42). However, their interpretation is not always straight forward. On one hand, when supplemented with selenium, GPx levels in ruminant erythrocytes increase, which is expected as GPx is a selenium dependent enzyme (43). These experiments interpret this result as an improvement in antioxidant status. On the other hand, there are greater levels of antioxidant enzymes in erythrocytes of dairy cows in the summer than in spring, which is due to increased heat stress (44). These increases are due to increased oxidative stress, as it is known that heat stress causes oxidative stress (44, 45). Due to these inconsistencies, we recommend implementing multiple markers of antioxidant status in order to assist with interpretation.

#### Intrinsic Non-enzymatic Defense Against Oxidative Stress

Non-enzyme antioxidants such as glutathione, uric acid, melatonin, bilirubin, polyamines, and metal binding proteins are also a part of the intrinsic oxidative stress defense system (41). While important, this review will not delve into detail on them. Mironczuk-Chodakowska et al. (41) provides a detailed review on non-enzymatic, intrinsic antioxidants. One example of non-enzymatic antioxidants is albumin, which is important to grazing ruminant health. Albumin is the major antioxidant in circulating blood, which is continuously exposed to oxidative stress (46). In ruminants, albumin has been found to be incorporated into colostrum and milk (47). Thus, albumin provides antioxidant defense in several biological fluids, such as blood, colostrum, and milk. In the following section we will go in depth on how a diverse diet providing biochemical diversity in plant secondary compounds can provide extrinsic antioxidant defense.

## Dietary Diversity and Antioxidant Defense

Extrinsic (exogenous or dietary antioxidants) defenses against oxidative stress come from food. These antioxidants include vitamins E and C and PSC, such as phenolics, terpenes and terpenoids. When offered an array of forages animals select and consume natural antioxidants at rates below toxic levels of intake (48, 49). Plant secondary compounds, especially phenolic compounds, have been shown to improve antioxidant status and reduce plasma levels of oxidative components. Phenolic and polyphenolic compounds (tannins and flavonoids, from terrestrial plants; phlorotannins, from aquatic plants [seaweeds]) can have free radical scavenging properties. Phenolic compound containing-extracts from the common daisy (*Bellis perennis L*.) showed free-radical scavenging activity of 2,2-Diphenyl-1-picrylhydrazyl *in vitro* (50). This ability was likewise demonstrated with isolated flavonoids from *Opuntia monacantha* (51). Additionally, Chakraborty et al. (52) extracted phlorotannins from three species of red seaweed (Division: *Rhodophyta*) and saw marked reductions in free radicals. This antioxidant activity can have remarkable effects on antioxidant status when applied to plants and animals (53, 54). Kannan et al. (55) reported increased antioxidant enzymes and reduced lipid peroxidation when sheep were treated with a seaweed extract and challenged with transportation stress. Milking goats provided tannins from sulla (*Sulla coronarium L*.) forage, had improved plasma antioxidant capacity (56). Sheep provided plant by-products (tomato pomace and grape skin) had upregulated transcriptional activity to genes that are involved in oxidant defense enzymes (57). When transition dairy cows were provided tannins from chestnut there were lower plasma and milk malondialdehyde (MDA; a marker of lipid peroxidation) and increased antioxidant enzyme activities in plasma and the liver (58). These experiments, and others, highlight the potential of PSC to improve antioxidant status, which would result in a better internal state and improve hedonic well-being of grazing ruminants.

### Plant Secondary Compounds as Antioxidants: Potential Modes of Action

Several modes of action exist for PSC, especially phenolic compounds, to exhibit antioxidant activity. One mode would be by providing antioxidant activity directly in bodily fluids and tissues. In order for this to occur, antioxidant PSC would need to be absorbed and incorporated into tissues (59). Evidence for this can be seen by the increased product quality, such as improved shelf life, color stability, flavor, and odor, from animals provided PSC seen by several experiments (59). One experiment provided sheep plant extracts, including rosemary (*Rosemarinus officinalis*), grape (*Vinis vitifera*), citrus (*Citrus paradise*), and marigold [*Calendula officinalis*; (60)]. It was found polyphenolic compounds, including condensed tannins from grapes, are catabolized to monomeric phenolics, become bioavailable, and were present in the blood of the sheep. It was also reported that naringin from the citrus extract was found in the plasma, which is contrary to what occurs in monogastrics (60). Another work reported that the ultraviolet-absorbing compounds in milk result from ingested phenolic compounds from forages [various hays, silages, and fresh pasture; (61)]. Additionally, when supplied tannins from sulla, goat milk was found to have greater phenolic compounds and total antioxidant capacity (56). These experiments indicate that antioxidant PSC such as phenolics can be absorbed from ruminant gastrointestinal tracts and incorporated into milk products thus improving product quality, but also potentially exerting nutraceutical, pharmaceutical, and prophylactic activity.

Plant secondary compounds have also been measured in meat products. When ewes were dosed rosemary (*Rosmarinus officinalis*) extract, their offspring were found to have increased phenolic compounds incorporated in their meat at slaughter (62). This incorporation in the tissue increased the antioxidant capacity of the meat (62). In a similar experiment, Nieto et al. (63) gave pregnant ewes either 0, 10, or 20% of their diet with distilled rosemary leaf and observed delayed lipid oxidation, odor, and flavor spoilage of their lamb's meat due to the additions. In another experiment, rosemary leaf distillate additions to pregnant ewes improved lamb meat quality characteristics (64). These results were corroborated when ewes were provided varying rates of thyme (*Thymus zygis ssp*.) leaves in their diet. Again, the antioxidant additions to their dam improved product quality and shelf life of the lamb meat (65). When lambs received a diet containing quebracho (*Schinopsis lorentzii*) tannins there was a 31.29 and 16.81% increase in total phenols and antioxidant capacity in the muscle compared to the control, respectively. The increased antioxidant status improved meat color stability (54). Similarly, when growing chickens were provided a by-product of the olive oil industry (semi-solid olive cake; “pate”), meat oxidative stability was improved and tyrosol and metabolites of hydroxytyrosol (phenolic compounds) were detected (66). These results support the mode of action for a direct antioxidant activity at the bodily fluids and tissue level by absorbed PSC and this interpretation has also been suggested by Vasta and Luciano (59).

Another potential mode of action for PSC is by providing antioxidant action in the gastrointestinal tract. As discussed above, the gastrointestinal tract is a major source of oxidants. This effect in livestock, to our knowledge, is largely unexplored. However, when sheep had long-term exposure to dietary heavy metals, it was found that there was oxidative damage to the gastrointestinal tract and concluded that lipid peroxidation was one of the mechanisms behind chronic heavy metal poisoning in ruminants (67). In humans, PSC, such as phenolics, have been found to alleviate or prevent gastrointestinal diseases such as ulcers (68). Evidence of antioxidant benefits of PSC in ruminant's gastrointestinal tract is lacking and requires further research; however, as stated above we speculate that the ruminant gastrointestinal tract is a major source of oxidants and postulate that antioxidant PSC would alleviate this production.

Finally, PSC have been found to regulate gene expression to alter antioxidant status. The nuclear factor erythroid 2-related factor 2 (Nrf2) has been identified as the leading transcription factor behind oxidative stress defense (69). Nuclear factor erythroid 2-related factor 2 reduces oxidative stress directly by increasing antioxidant enzyme activity, regenerating oxidized cofactors (e.g., GSSG to GSH), synthesizing these reducing factors (e.g., GSH), and by increasing expression of antioxidant proteins (69). Plant secondary compounds have been shown to activate Nrf2, resulting in increased antioxidant enzymes in farm animals (70). An *in vitro* experiment on bovine mammary epithelial cells showed potential of tea polyphenolics to reduce oxidative stress when challenged by hydrogen peroxide, and that these results were due to upregulation of Nrf2 (71).

Oxidative stress is an important aspect of ruminant management. Reactive oxygen metabolites are both necessary for normal physiological functioning (e.g., redox signaling) but also, when produced at levels that outpace the animal's defense system, can cause negative effects after they damage various biological molecules. The defense system in place for ruminants to handle oxidants are intrinsic antioxidant enzymes and non-enzyme antioxidants, but also extrinsic dietary antioxidants. Plant secondary compounds, which can be commonly found in many forages used in grazing ruminant production systems, provide an interesting opportunity to manage oxidative stress in grazing ruminants as they have several modes of actions. They can remove oxidants in the gastrointestinal tract, after absorption in the small intestine, by being incorporated into milk and tissues, and by regulating gene expression.

### Physiological Stress and Hedonic and Eudaimonic Well-Being

Physiological stress is the hormonal response that an organism experiences in response to a stressor, whether abiotic or biotic. Physiological stress manifests itself in the “fight or flight” response in organisms (20). This response is elicited by the release of glucocorticoids (GC). Glucocorticoids, such as cortisol, have been studied as a marker of animal welfare with less cortisol acting as a marker for positive welfare (20). Abiotic stressors include climatic events (e.g., heat stress). Biotic stressors are elicited from the animal's peers, predators and other animal species, animal handling (72), and, more recently suggested, dietary monotony (4, 5, 8, 73). Dantzer and Mormède (20) reviewed the causes of physiological stress and physiological pathway, from stress perception to hormonal responses. In brief, following the experience of a stressor, glucocorticoids (GC) are released following the hormonal cascade from the hypothalamic-pituitary axis (20). In essence GC prepare the animals for the “fight or flight” through several metabolic responses. These include increased gluconeogenesis, reduced glucose uptake by the periphery, suppress insulin, and mobilize energy stores. Additionally, GC can alter behavior and elicit anxiety behavior [e.g., stereotypies; (20)].

Historically, objective assessment of animal welfare has been done by measuring GC in the blood (20). As animal handling to take the blood sample causes a stress response, it has been suggested that fecal cortisol metabolites (74) or hair (75) cortisol levels are more accurate. While cortisol is the most predominant biomarker of welfare, there are several other markers available [see (76) for a recent review]. However, most methods of measuring welfare would only provide insight on the negative state of the animal and it is often assumed that less cortisol provides insight into positive welfare, which may not always be the case (77). This necessitates research into objective markers of positive welfare. Some markers of positive welfare that have been suggested are vocalizations, measurements of neurotransmitters such as endorphins and dopamine, and hormones like oxytocin and serotonin (77, 78). While physiological stress and negative welfare may often be negatively correlated with positive welfare, it is time for the development of standardized methodologies for measuring positive states of animals.

Ethical management of animals has been predominately based upon the “five freedoms.” These include the freedom from (1) thirst, hunger, and malnutrition, (2) discomfort and exposure, (3) pain, injury, and disease, (4) fear and distress, and (5) freedom to express normal behavior (79, 80). All of these freedoms relate to hedonic well-being, with the exception of freedom 5. Hedonic well-being is based upon pleasure and comfort seeking (10). More recently, there has been a call in the literature and from the public for animals to have “A Life Worth Living” (81) or “the Good Life” (12). As such, animal welfare concerns are moving away from merely ensuring that animals are provided with the opportunity to perform (by ensuring that they have adequate nutrition, freedom from fear, sickness, and discomfort), to ensuring that they have a life worth living (at least in terms of anthropomorphic understanding of “worth”). This appeals to the eudaimonic theory of well-being. For further readings find (81) and then the invited response (79).

Under eudaimonic theory, well-being is a process and not a state. It stems from the pursuit of a good life through individual choices (10). Much of what is known about eudaimonic well-being comes from philosophy, but recently scientific evidence has been gathered to support this theory using human subjects (82). In grazing animals, more research is required to investigate eudaimonic well-being and we believe that experiments centered around providing choices are particularly needed. Evidence for support of Eudaimonia in livestock has been shown in zoo animals. Giant pandas had lower urinary cortisol when they were provided a choice between two environment enclosures compared to pandas who were only allowed access to the exhibit environment. As the added enclosure area was less enriched, the choice group spent most of their time in the exhibit area, and there were no differences in active time it was concluded that the enhanced animal welfare was derived from the ability of the animals to choose (15). In foraging ruminants, some support for *telos* and eudaimonic well-being may be seen in grazing personalities (8). One example of grazing personalities was described by Bailey et al. (14), who found that there are cattle who prefer to graze in the flat low fields, termed bottom dwellers, and cattle who prefer to climb mountainous areas for grazing, termed hill climbers. It was found that these specific grazing personalities were related to genetic markers (14). This is interesting as *telos* has been described as intrinsic in the genetic coding of animals (12). Thus, we hypothesize that individual animal's personalities, including grazing personalities, provide insight to individual animal's *telos* and thus provide evidence of eudaimonic well-being. Additionally, we speculate that this theory will apply to livestock and that enhanced welfare from dietary diversity both facilitates and is evidence to support this theory, which is discussed further below, even though separating welfare enhancement of dietary diversity between hedonic and eudaimonic well-being is difficult.

## Linking Dietary Diversity and Physiological Stress

Choice is a key concept in the eudaimonic theory of human well-being, with the overarching concept being to pursue a life of fulfillment of one's true nature, or *telos*, with choices being important in this pursuit (10). Eudaimonia often stands in contrast to the hedonic theory of well-being, which considers contentedness as the sum of positive and negative affective states, i.e., emotions (10) and which has been a primary focus of studies of animal welfare (13). However, recently several works have explored the effect of choice on livestock welfare. Catanese et al. (83) gave lambs either a choice of different foods contrasting in protein: energy ratios (diversity) or all of those foods provided in a total mixed ration (monotonous). It was found that when animals were allowed to choose, they had lower cortisol, than their counterparts (83). Villalba et al. (73) has also shown similar results in lambs. When lambs were offered a four-way choice between foods which were diverse in nutrient composition or in PSC, there was lower plasma cortisol concentrations compared to lambs who received a monotonous diet of all food options. Manteca et al. (4) and Villalba et al. (5) reviewed dietary choice as an important aspect of animal welfare and related it to animals being able to balance their own nutrients to meet individual requirements through nutritional wisdom, and also to balance intake of PSC so that they can experience their benefits (e.g., reduced gastrointestinal parasites) without experiencing toxicities, which all relate to hedonic well-being. Additionally, hedonic well-being is partly responsible for controlling feeding behavior in ruminants (84). While these are likely true, dietary diversity may also reduce stress merely by providing the animals a choice, if the Eudaimonic theory of well-being can be applied to livestock. Additionally, we postulate that dietary diversity likely enhances both hedonic and eudaimonic well-being (Figure 1), as it has been found that these two mental well-being states contribute to welfare in different and overlapping ways (85).

As mentioned previously, dietary choice allows animals to consume PSC at amounts that provide benefits, while staying below the threshold at which negative effects occur. In one experiment, sheep faced either no challenge (received saline injection), an adrenocorticotropic hormone (ACTH) challenge only, or an ACTH challenge plus one of four PSC (containing polyphenols) products (86). Sgorlon et al. (86) examined global mRNA expressions in sheep blood in response to the blood cortisol levels, which resulted from the ACTH challenge. As expected, ACTH treatment caused increased cortisol production after 3 and 51 h. While the sheep that received plant secondary compounds did not experience reductions in cortisol production, it was determined that the PSC altered the molecular signature produced as a result to increased cortisol. Overall it was determined that while ACTH challenge reduced gene expression involved in immune response, when provided PSC products, this effect was attenuated, but the results were dependent upon the product used (86). Thus, PSC may improve the response that animals have to physiological stressful events.

There is a known relationship between diet and the composition of ruminal microorganisms. For example, Tapio et al. (87) in a 4 × 4 factorial, fed dairy cows two levels of forage-to-concentrate (high, 35:65; low, 65:35) with either 0 or 50 g sunflower oil/kg diet dry matter. It was determined that there were taxa abundance changes and microbial interactions that were diet specific. Similar results have been seen in cows fed alfalfa or triticale forages (88). The composition of ruminal microorganisms can likewise influence the ruminal degradability of feeds, but also fermentation end products as specific microorganisms produce different fermentation end products, as they often fill dietary niches (89, 90). As the majority of energy available for ruminants to use for metabolism come from fermentation end products [~70% in ruminants; (91)] and the ruminal microbiome composition determines the types of fermentation end products produced, several experiments have shown a link between the rumen microbiome composition and feed efficiency traits (92–94). Therefore, the ruminal microbiome is an important aspect of ruminant nutrition and is dependent on diet.

Relatively recently, there has been much work on how microbial fermentation products (e.g., volatile fatty acids) can alter mood, behavior, and subsequently physiological stress, mental well-being, and welfare, through what is termed the microbiota-gut-brain axis. Much of this work has been done with humans. In a review, it was concluded that the gut microbiota in humans can communicate with the central nervous system, subsequently altering mood, cognition, and emotions (95). Likewise, the microbiota-gut-brain axis has been shown to influence behavior (anxiety and social) and memory capacities in non-ruminant livestock (96). Microbial fermentation products are known to alter feeding behavior in ruminants and to provide positive postingestive feedback to the animals, thereby providing positive emotions and influencing the preference for specific foods. For instance, when different flavors were offered to sheep and associated with a low or high addition of exogenous propionate (a glucogenic volatile fatty acid), it was found that at lower additions ruminants developed a dietary preference for the conditioned flavor, whereas at the higher addition the sheep developed aversions to that flavor (97, 98). This relationship makes intuitive sense as the volatile fatty acids provide 70% of the caloric requirements of ruminants (91). In humans and other mammals the microbiota-gut-brain axis has been shown to influence behavior, mood, and emotions, while the only predominant link shown in ruminants is its effect on feeding behavior (96). The availability of information on the microbiome-gut-brain axis and its effect on non-eating related behaviors in ruminants may be lacking because fermentation products represents a much larger contribution to their nutritional requirements than humans or other farm animals. For example, while volatile fatty acids contribute 70% of energy for metabolism in ruminants, it only accounts for ~10% for humans, 25% for pigs, and 30% for rabbits and horses (91). However, there is still a need to determine how the ruminal and hind-gut microbiome may alter non-eating related behaviors in ruminants.

Dietary diversity would likely influence the ruminal microbiome composition, which in turn may influence the host animal's mood, emotions, and welfare and would influence dietary preference and dry matter intake. A review of the human literature concluded that a diverse diet would supply a wide range of substrates for the microbes to ferment in the gut, which would promote a more diverse microbiome (i.e., microbial species richness). This diverse gut microbiome was suggested to be more adaptable to disruption (99). It is known that diet formulation alters the ruminal microbial species richness. For instance, when grain based diets are fed to ruminants they have a less diverse microbiome compared to forage based diets (100) and differences have also been shown when cows were fed different forages (88). However, to our knowledge, there is no information available for how dietary diversity may influence the ruminal microbiome's species richness. Therefore, there is a need to determine how dietary diversity may influence the microbiome of grazing ruminants. Additionally, while it is known that microbial fermentation products can alter dietary preference and intake behavior in ruminants, there is a lack of knowledge on if the fermentation products could influence other behaviors, and subsequently mental well-being and welfare, in ruminants. However, based on experimentation with other mammals (humans, rats, etc.), there is a strong possibility for dietary diversity to alter the microbiome, mood, and emotions when provided to ruminants.

Grazing ruminant mental well-being and nutrition are closely linked. Hedonic well-being influences voluntary feed intake through changes in opioid, cannabinoid, and the GABA systems, thus providing a reward response and influencing how ruminants like a specific food [(84); Figure 1]. By providing dietary diversity, animal well-being may be improved in several ways. One is through improved eudaimonic well-being, by providing the animals with choice, thus allowing control over the animal's environment and the expression of individuality. Another means is through improved hedonic well-being by enhanced internal state by improved nutritional status. Additionally, PSC consumed at an appropriate level, which is allowed by dietary choice, have direct effects on the response that the animal has to physiological stress. Finally, dietary diversity may alter the microbiome-gut-brain axis, which has been shown to alter the mental well-being of other mammals.

## Nutrition as Effected by Dietary Diversity

Ruminants have evolved in ecosystems where dietary choices abound and where they were able to select plants differing in PPC and PSC so that they could consume a balanced diet that met their needs for nutrients, medicine, and prophylactics (9). Dietary diversity and allowing animals to choose from an arrangement of feedstuffs to meet their own requirements is not a new concept (9). As ruminant nutritionists, requirements are typically assessed and food offered to meet those requirements for an average animal. However, if we expect dietary requirements to follow a normal distribution, a small number of animals would be “average” and thus, ~50% of animals will be fed diets that under supply nutrients and around 50% will over ingest nutrients (1, 2). Therefore, lack of dietary choice may result in individual dietary imbalances. These nutrient imbalances may lead to incidental restriction or augmentation (6). Incidental restriction is a reduction in intake due to negative post-ingestive feedbacks as a result of over consuming specific nutrients and incidental augmentation occurs when animals over ingest nutrients in order to meet their requirements for nutrients that are in lower concentrations in the diet (6). The differences between individual animals are a result of variations in physiological and morphological differences (1) and also due to individual personalities (101). In a grazing context, Parsons et al. (102) found that, overall, sheep prefer to mix their diet and that their dietary preferences change across the day, influenced by sward characteristics and their previous diet. Parsons et al. (102) measured preference by video-recording grazing location (i.e., forage species) and calculating forage intake from previously established intake rates of the respective forages. Individual animals vary greatly with regard to selection of dietary components within and between meals. It's because of these differences of individual animal preference and selectivity that common management goals aim to reduce sorting and selectivity by cattle fed “total-mixed rations” (103). These management goals often involve adding liquids (e.g., molasses or water) to the mixed rations (103). Interestingly several works have shown these management strategies actually encourage feed sorting and reduce dry matter intake and this has been related to the lower dry matter diets having greater temperatures resulting in increased spoilage (104, 105).

The difference in individual animal selection is likely due to individual variation in the internal-state and post-ingestive feedback mechanisms that govern intake. This means that providing animals choice in the dietary constituents, rather than offered as a “total-mixed ration” formulated for the average animal or a non-functional mixed sward (mixed swards planted in a way that inhibit selection) may allow animals to choose from the dietary constituents in order to meet their respective requirements (8). Ruminant producers offering livestock high concentrate diets prefer to feed total mixed rations for ease of management and to reduce risks for negative health problems (e.g., ruminal acidosis and laminitis). However, it has been suggested that by offering choice ruminants can alter eating patterns to account for the later concern (2) and this has been supported by experiments where grain was offered at free choice and pH was measured (106, 107). While there has been much research on feeding total mixed rations in the last 60 years [see (108)], there is surprisingly few experiments which have compared total mixed rations compared to the dietary constituents offered as choice, but many of those that have, found choice to be superior. In an early experiment, reduced dry matter intake, similar performance, and improved feed efficiency were observed when dairy cows were offered forage and grain separately as opposed to being provided a total mixed ration (109). Another experiment conducted in feedlot fed steers provides further evidence for this hypothesis (2). Cattle were offered either a total-mixed ration or the components of the total-mixed ration offered individually. It was found that the diet selected by cattle varied tremendously between animals, but also within animals across days. The cattle offered choice consumed less feed, had similar performance, and lower cost of gain compared to the total mixed ration treatment (2). A separate experiment conducted by the same laboratory with growing sheep found that when lambs were provided choice between three iso-caloric and iso-nitrogenous diets, they had greater dry matter intakes, performance, and feed efficiency, and less cost-of-gain compared to lambs offered only one of the three diets (110). These experiments have been corroborated by other laboratories. When lactating goats were offered choice they consistently consumed less dry matter comparable milk productions compared to their total mixed ration counterparts (111). It is important to note that some experiments have found choice and total-mixed rations to be not significantly different (106, 107) or for total-mixed rations to be superior (112). Likewise, others contend that ruminants possess poor internal wisdom and that they are unable to select diets according to their nutrient requirements (108). These different findings and conclusions may be due to the differences in the dietary options provided. If dietary constituents are not divergent enough in nutritive composition, then animals may not be able to select diets tailored to their specific individual nutrient requirements. Several experiments across multiple species and production settings have shown choice to improve feed efficiencies (either by reducing intake while maintaining intake or by increasing intake and performance) compared to offering a total-mixed ration, which were formulated to be optimal for the average animal. This is clear evidence for the importance of dietary choice as a means for meeting the individual requirements and avoiding incidental restriction or augmentation of intake.

## Linking Oxidative, and Physiological Stress

In humans, a link between oxidative status (metabolic stress) and physiological stress has been suggested and reviewed, with an apparent vicious cycle where physiological stress increases metabolic stress, which in turn increases physiological stress, etc., resulting in telomere shortening and aging (113–115). This may be especially true in scenarios of chronic stress (115, 116). Aschbacher et al. (116) explored the effect of chronic stress and perceived acute stress and found that there was significant oxidative damage when chronically stressed people experienced a perceived stressor. Chronic stress occurs when there are relatively high levels of glucocorticoids circulation in the blood stream for a prolonged period of time. Chronic stress has been linked to health problems in humans and animals. Several works have reported increased oxidative stress as a possible mode of action behind the cost of chronic stress (117). Orzechowski et al. (118) explored how rat's antioxidant status and oxidative stress changed when challenged with dexamethasone (a synthetic GC; 2-mg/kg of body weight/d). It was found that treatment with dexamethasone decreased blood and muscle glutathione, reduced SOD activity, and increased malondialdehyde [measured by TBARS; (118)]. A meta-analysis by Costantini et al. (117) concluded that GC were significantly associated with oxidative stress and that there were different magnitudes of effects according to tissue, sex, and age. Therefore, physiological stress increases oxidative stress in livestock and other mammals.

There is little direct evidence to link physiological stress and oxidative stress and their subsequent consequence in livestock. The experiments that have explored these relationships generally compared animals before and after a physiologically stressful event. In one experiment, 105 crossbred steers where transported for 19 h and 40 min. This stressful event significantly reduced serum antioxidant capacity and increased malondialdehyde (marker of oxidative stress). It was found that calves with more incidence of bovine respiratory disease also had higher oxidative stress after transportation (119). Other common management practices, which are known to be stressful to animals have been linked to oxidative stress. After sheep were shorn, there were greater circulating malondialdehyde (marker of lipid peroxidation) concentrations than before shearing (120). Finally, malondialdehyde was likewise increased after cattle were dehorned (121). These experiments provide evidence that physiological stress increases oxidative stress in livestock. However, there is less evidence to show that dietary antioxidants can reduce physiological stress. One experiment challenged sheep with injections of ACTH and found that a treatment group provided with supranutritional antioxidants (Vitamin E and Se) had lower circulating cortisol compared to their non-supplemented cohorts (45). Some recent works have shown a positive correlation between isoprostanes, which results from oxidant conversion of arachidonic acid, and cortisol. These experiments explored the effects of a non-steroidal anti-inflammatory drug on reproductive performance of cattle (29, 30). As mentioned previously, isoprostanes result from the peroxidation of arachidonic acid by oxidants and it has been suggested that they are the pathophysiological mediators of oxidative damage (28). A positive correlation between cortisol and isoprostanes provides direct evidence and a potential mode of action for a link between oxidants and physiological stress. However, the relationship between physiological and oxidative stress is an area that requires further investigation in livestock, but there is evidence that improving oxidative status may allow the animal to better recuperate from the stress and reduce subsequent negative effects.

## Linking Oxidative and Nutritional Status

Metabolic disorders seen in transition dairy cows provides excellent insight into how oxidative stress can be effected by the nutritional status of the animal. Bernabucci et al. (22) followed 24 cows with different body condition scores (BCS) across the transition period (± 30 d at calving). It was concluded in this experiment that oxidative status was related to energy status and that cows with greater weight loss over this period experienced greater oxidative stress (22). This positive relationship between energy demand and negative energy balance and oxidative stress has been shown in several experiments. For example, milk yield is positively correlated to markers of oxidative stress in dairy cows [malondialdehyde, (122) and hydroperoxides (123, 124)]. While most of the experiments focused on nutrition and oxidative stress in dairy cows, the relationships are applicable across ruminant species. For example, when lambs were fed 70 or 80% of their metabolizable energy requirements, they had higher plasma malondialdehyde levels than when lambs were fed 100% of their requirements (125). Level of energy intake is not the only way oxidative status is influenced by nutrition, in fact source of energy can have impacts. When lambs were fed high fat, there was an increase in blood superoxide dismutase levels and glutathione concentrations. This was attributed to increased fatty acid oxidation which would stimulate the production of oxidants (126). Nutritionally related disorders such as subacute ruminal acidosis can also induce oxidative stress. Guo et al. (127) found that when dairy cows were induced with subacute ruminal acidosis, there were lower plasma levels of total antioxidant capacity and higher glutathione peroxidase activity and malondialdehyde concentrations. Nutritional status and oxidative stress are intimately linked. Oxidative stress can be influenced by previous level of nutrition, current energy intake, source of energy, and also nutritional related diseases.

As mentioned above, diet can greatly influence the composition of the ruminal microbiota. Further, we discussed above how microbial fermentation products can influence physiological stress and welfare. As physiological stress and reduced welfare will likely lead to oxidative stress, the effect that dietary diversity may have on the ruminal microbiota, or even the hind-gut microbiota, may provide another mode of action for dietary diversity to reduce physiological and oxidative stress. However, this requires further investigation. Additionally, microorganisms have been directly linked to reductions in oxidative stress. For instance, cattle had less antioxidant activity in their ruminal fluid and in their plasma when they were defaunated (removal of protozoa) compared to their faunated cohorts (39). Also, steers placed in a feedlot had less glutathione peroxidase activity (indicating less oxidative stress) and a greater blood antioxidant level when provided a lactobacillus fermentation product compared to the control steers (128). Thus, dietary diversity may alter mood and behavior, thereby influencing mental well-being and welfare, indirectly by altering the ruminal microbiota composition and also may directly reduce oxidative stress.

## Concluding Remarks

This review has covered oxidative stress, physiological stress, and nutritional status, which are areas of animal science that are important to both producers and consumers. Further, we have provided links between these three areas and have described how dietary diversity links the three. In conclusion, there is evidence to support how dietary biochemical diversity (provided through taxonomical diversity) can reduce oxidative stress directly by providing plant secondary compounds as natural dietary antioxidants and indirectly by reducing physiological stress, which we have reported evidence to influence oxidative status. Additionally, the antioxidant benefits of plant secondary compounds may improve the metabolic response the animal has to physiological stress and therefore improve the response to the perceived stress. Dietary diversity may improve eudaimonic well-being merely by allowing animals to make choices, and thus we postulate that this theory of well-being applies to livestock. Further, diverse diets may alter the microbial-gut-brain axis, which in humans and some non-ruminant farm animals has been shown to alter cognition, mood, emotions, and behavior, as well as dietary preference and eating behavior in ruminants. Finally, dietary choice allows animals to take advantage of differences in plant primary compounds to meet individual animal requirements and thereby improve nutritional status. Improved nutritional status can subsequently have beneficial impacts on oxidative stress by reducing energy store mobilization and physiological stress by improving hedonic well-being. Physiological stress, oxidative stress, and nutritional stress are intimately linked (Figure 1) and dietary choice compared to monotony may simultaneously improve all of these items directly and indirectly, resulting in marked improvements in the foraging animal's nutritional status, health, mental well-being, and ultimately their welfare. We conclude that dietary diversity reduces stresses while enhancing hedonic and eudaimonic well-being in ruminant livestock.

## Author Contributions

MB was responsible for the majority of formulating content and writing the submitted manuscript. PG is MB adviser and assisted in writing, formulating content of manuscript, proofreading, and suggesting edits.

### Conflict of Interest

MB received funding from Agrisea Ltd (Paeroa, New Zealand) and Callaghan Innovations (New Zealand) to support his Ph.D. studies. The remaining author declares that the research was conducted in the absence of any commercial or financial relationships that could be construed as a potential conflict of interest.
